# What has a greater influence on adolescent mental health: BMI or self-efficacy? Evidence from a Peruvian sample

**DOI:** 10.1186/s40359-025-03055-7

**Published:** 2025-07-02

**Authors:** Daniela Huanca-Cachicatari, Raquel Navarro-Carrasco, David Javier-Aliaga, Bertha Chanducas Lozano, Yaquelin E. Calizaya-Milla, Jacksaint Saintila

**Affiliations:** 1 School of Human Nutrition, Faculty of Health Sciences, Universidad Peruana Unión, Lima, Peru; 2 Research Group for Nutrition and Healthy Behaviors, School of Medicine, Universidad Señor de Sipán, Chiclayo, Peru

**Keywords:** BMI, Self-efficacy, Mental health, Adolescents

## Abstract

**Background:**

Mental health in adolescents is a public health issue with both immediate and long-term implications. Several studies have demonstrated that Body Mass Index (BMI) and self-efficacy are associated with this issue, although they have generally been examined independently. Therefore, the aim of this study was to determine the predictive role of BMI and self-efficacy in mental health in a sample of Peruvian adolescents.

**Method:**

The research was non-experimental and predictive in nature. The sample consisted of 343 students (49.6% male and 50.4% female), aged 12 to 17 years (M = 14.71; SD = 1.08), from a public educational institution in Metropolitan Lima, Peru. The sample was selected through non-probabilistic, purposive sampling. The General Self-Efficacy Scale (GSE) and the Mental Health Inventory (R-MHI-5) were used, and BMI was measured using Quetelet’s formula.

**Results:**

The results showed a significant relationship between BMI and mental health, as well as between self-efficacy and mental health (*p* < 0.001). Additionally, no statistically significant differences were found between BMI, self-efficacy, and mental health according to gender and age (*p* > 0.05). Moreover, multiple regression analysis revealed that the predictor variables BMI and self-efficacy explained 25.1% (*adjusted* R^2^) of the variability in adolescent mental health. The standardized coefficients (β) indicated that self-efficacy (0.452) had a greater impact on the prediction of mental health than BMI (-0.202).

**Conclusions:**

These findings demonstrate that self-efficacy, understood as adolescents’ personal beliefs about their ability to control their lives and solve problems, has a more significant impact on their mental health than their body mass index (BMI). Therefore, it is recommended that educational and public health interventions aimed at improving mental health adopt a holistic approach that integrates both physical and nutritional components (BMI) as well as psychological components (self-efficacy).

## Introduction

Adolescent mental health has emerged as a significant public health concern [1]. This variable is defined as a state of well-being in which an individual realizes their potential and experiences reduced psychological distress [2]. The World Health Organization (WHO) has reported that one in seven adolescents, aged 10 to 19 years, globally experiences mental health issues [3]. In the U.S., it has been reported that approximately 20% of children and adolescents aged 3 to 17 years suffer from a mental, emotional, developmental, or behavioral disorder [4]. In Peru, it is estimated that one in ten children is affected by a mental disorder [5]. Moreover, poor mental health has detrimental consequences for adolescent well-being [6]. Mental health problems in school settings can lead to poor adjustment, concentration difficulties, reduced academic performance, increased risk of school dropout or expulsion, problematic social relationships, and a higher incidence of health risk behaviors, such as substance use [7]. Studies also indicate that the deterioration of adolescent mental health can lead to anxiety, depression, psychological stress, and, in severe cases, suicide [8].

Elevated Body Mass Index (BMI) values significantly affect overall health, cognitive development, and, in particular, the mental health of adolescents [9–11]. BMI, as proposed by the World Health Organization (WHO), is an index calculated using the Quetelet formula to evaluate the appropriateness of an individual’s body mass [12–14]. According to WHO, the global prevalence of obesity among children and adolescents aged 5 to 19 years increased fourfold between 1990 and 2022, rising from 2 to 8% [15]. In many developing countries, more than half of adolescents fail to reach their full growth potential due to insufficient nutrition and inadequate dietary habits [16]. Moreover, an inadequate BMI is linked to a range of psychological and physical issues in adolescents. Studies show that those struggling with obesity are more likely to experience psychological problems, such as low self-esteem, poor health-related quality of life, depression, anxiety, behavioral disorders, bullying, lack of motivation, poor academic performance, and impaired cognitive development, among others [11, 17]. Similarly, the nutritional status of adolescents poses a significant risk to long-term cardiovascular health, the development of noncommunicable diseases, and immune function [18]. Finally, scientific literature has demonstrated that malnutrition, overweight, and obesity are significant risk factors for mental health issues in adolescents [19].

Self-efficacy has also been recognized as a key factor in understanding mental health [20, 21]. This construct is defined as an individual’s belief in their ability to organize and execute actions necessary to achieve specific goals [22]. Higher levels of self-efficacy are associated with an increased sense of subjective well-being (happiness) and improved psychological well-being [23, 24]. Conversely, research has demonstrated that low self-efficacy in adolescents leads to various negative outcomes. For instance, Mazlominezhad et al. suggest that low self-efficacy results in individuals feeling incapable of controlling events, actions, and emotions in their lives [25]. Additionally, other studies have shown that low levels of self-efficacy are associated with emotional difficulties, including symptoms of depression, anxiety, and low self-esteem [26, 27]. Finally, researchers have identified self-efficacy as a key protective factor for mental health [6, 23, 28].

The biopsychosocial model is a theoretical framework that integrates biological, psychological, and social factors to understand health and disease. Proposed by George L. Engel in 1977, this model challenges the reductionist perspective of the biomedical approach, acknowledging that an individual’s well-being results from the interaction of multiple factors [29]. Furthermore, current scientific literature supports this approach in the treatment of various diseases, recognizing that an exclusively biological perspective is insufficient to address the complexity of interactions between the mind, body, and environment [30]. The WHO also endorses this model, recognizing the importance of a holistic approach in promoting global health [31]. In the context of adolescent mental health, the biopsychosocial model is particularly valuable for understanding the relationship between body mass index (BMI) and self-efficacy in relation to mental health. BMI, as a biological indicator, may have a significant impact on adolescent mental health. However, self-efficacy, as a psychological factor, plays a pivotal role in shaping how adolescents cope with stress, which subsequently affects their mental well-being. Thus, the biopsychosocial model facilitates the integration of these variables, providing a holistic framework for understanding adolescent mental health.

The relationship between self-efficacy and mental health in adolescents has been consistently emphasized in the literature, with studies demonstrating a positive and significant correlation between these two constructs [6, 23, 24, 26, 32, 33]. On the other hand, only a limited number of studies have explored the relationship between BMI and mental health in this age group [11, 19]. However, no studies have been identified that simultaneously analyze the relationship between these three variables, particularly within the Peruvian context. This gap in the literature highlights the need for further research to address this important area.

This study is justified by the need to understand how self-efficacy, BMI, and mental health interact in the Latin American context, and particularly in the Peruvian context. This exploration is relevant considering that cultural, social, and economic factors specific to this region may influence how these variables relate to and affect the mental health of adolescents. Socioeconomic factors, such as inequality, poverty, and limited access to health services [34, 35], and cultural factors, such as family dynamics, which are a key resource for the positive development of Latino individuals [36], can influence the mental health of adolescents. This approach could offer valuable insights for the development of more effective, culturally tailored interventions aimed at promoting psychological well-being among Peruvian adolescents, taking into account both their self-efficacy beliefs and physical health status. Thus, the main objective of this study is to determine the predictive role of BMI and self-efficacy on mental health in a sample of Peruvian adolescents. Specifically, the following objectives are proposed: (1) to conduct a descriptive and comparative analysis of BMI, self-efficacy, and mental health according to gender; (2) to conduct a descriptive and comparative analysis of BMI, self-efficacy, and mental health according to age; (3) to determine the correlation between BMI, self-efficacy, and mental health; (4) to determine the predictive role of BMI on mental health; and (5) to analyze the predictive role of self-efficacy on mental health.

## Methods

### Methodological design

This research adopted a quantitative approach with a non-experimental, cross-sectional, and predictive design. According to the classification proposed by Ato et al. [37], the predictive design belongs to the associative strategy. This type of design is particularly suitable when the purpose of the study is to anticipate the behavior of a dependent variable, in this case mental health, based on one or more independent variables, such as BMI and self-efficacy [37, 38].

### Participants

The sample consisted of 343 adolescents in grades two through five from a public school in Metropolitan Lima, Peru. Participants were selected through non-probability convenience sampling, based on inclusion and exclusion criteria established by the researchers [38]. However, the sample size criteria required for a multiple linear regression model were taken into account. Using the statistical software G*Power 3.1.9.7 [39], it was determined that a minimum of 107 participants was sufficient to detect effects with a significance level of α = 0.05, a statistical power of 0.95, a moderate effect size (f² = 0.15), and two predictors. However, it was decided to use a larger sample (*n* = 343). The decision to work with a larger sample than initially calculated was due to the high response rate in the intermediate grades, which strengthened the statistical power of the study.

Data collection was conducted in April 2024. The instruments were administered in person, with each adolescent taking approximately 7 to 10 min to complete the questionnaires, while anthropometric measurements (weight and height) were taken in an estimated time of 4 min. Participant responses were anonymous, and participation was voluntary. Initially, a total of 395 high school students were contacted through coordination with the institution’s administrators. Of these, 42 students were excluded for not meeting the inclusion criteria (e.g., not having a signed informed consent form, presenting diagnoses of depression or anxiety, being outside the age range of 13 to 17 years, not being enrolled in grades 2 through 5 of secondary education, incomplete questionnaires, being over 18 years old, or not having Peruvian nationality), and 10 students declined to participate voluntarily. The final sample consisted of 343 adolescents, of whom 65 were in the fifth grade. It is worth noting that the response rate was lower in this latter group, likely due to their limited availability for academic reasons, such as preparation for graduation.

Finally, the study was reviewed and approved by the Research Ethics Committee of the Faculty of Health Sciences at Universidad Peruana Unión, Peru (2024-CEB-FCS-UPeU-No. 186). In addition, the study adheres to the ethical principles established in the Declaration of Helsinki.

**Table 1:** Sociodemographic characteristics of the sample (*n* = 343)

		*n*	%
Age (M ± SD)		M = 14.71	DS = 1.08
Sex	Male	170	49.6
	Female	173	50.4
School grade	Second	100	29.2
	Third	84	24.5
	Fourth	95	27.7
	Fifth	64	18.7

Note. M = Mean; SD = Standard Deviation

Table 1 summarizes the sociodemographic characteristics of the sample, which consists of 343 adolescents. The mean age of the participants is 14.71 years, with a standard deviation of 1.08. The gender distribution is nearly equal, with a slight female predominance (50.4%). Regarding educational grade, the adolescents are fairly evenly distributed across second, third, and fourth grades, with fifth grade being the least represented (18.7%).

### Measuring instruments and equipment

#### General Self-efficacy scale (GSE)

The General Self-Efficacy Scale (GSE), developed by Schwarzer and Jerusalem in 1995, is a widely used instrument for measuring individuals’ belief in their ability to cope with a variety of difficult demands in life [40]. It is a unidimensional scale composed of 10 items, using a 4-point Likert-type format: always [4], almost always [3], almost never [2], and never [1]. The scale aims to assess an individual’s belief or confidence in their ability to perform new or challenging tasks and cope with adversity across various domains of human functioning. Reliability studies conducted in 23 countries reported Cronbach’s alpha values ranging from 0.76 to 0.90, with most values in the high 0.80 range [41] [36].

GSE was validated for the Peruvian context by Flores and Lysaytan in 2023 [42]. The structural validity, assessed using confirmatory factor analysis, yielded global goodness-of-fit indices, with an SRMR of 0.042 and an RMSEA of 0.079. In terms of comparative fit, a CFI of 0.92 and a TLI of 0.90 were observed, while for the parsimonious fit, an AIC of 8311 was obtained. Additionally, the Omega and Alpha coefficients were 0.84 and 0.85, respectively. Finally, the reliability for this study, as measured by Cronbach’s alpha, was 0.883.

#### Mental health scale (R-MHI-5)

The Mental Health Inventory (R-MHI-5) was developed by Berwick et al. [43]. The R-MHI-5 is a tool designed to assess mental health in both adolescents and adults. This inventory evaluates mental health through two approaches: the first assesses the presence of psychological well-being (items 2 and 4), while the second measures the absence of psychological well-being through reverse-scored items (items 1, 3, and 5). Together, these items reflect the individual’s overall state of well-being. The inventory consists of 5 items, with response options on a Likert scale ranging from never (0), sometimes [1], often [2], to always [3], and it is a unidimensional instrument.

The R-MHI-5 was validated by Rojas-Mendoza et al. [44]. The confirmatory factor analysis results (CFI = 0.99, TLI = 0.99, SRMR = 0.04, RMSEA = 0.101) and reliability coefficients were considered adequate, with values above 0.70. Similarly, in the present study, the reliability measured by Cronbach’s alpha coefficient was 0.723. In addition, the bifactor analysis conducted allowed for an assessment of the instrument’s structural validity, identifying a general factor that explains the majority of the common variance, along with specific factors that provide additional information (CFI = 0.983, TLI = 1.000, SRMR = 0.020, RMSEA = 0.000). These findings support the use of the total score as a valid indicator of mental health in adolescents.

#### Body mass index (BMI)

The BMI of the adolescents was determined through anthropometric measurements of weight and height. Weight was measured using a Tanita (Baby/Mon) model 1582 digital scale, with a minimum capacity of 40 kg and a maximum capacity of 120 kg. Height was recorded with a standard measuring rod, with a maximum height of 170 cm. BMI was then calculated using the Quetelet formula: weight (kg) / height (m²).

### Statistical analysis

Before proceeding with the main analyses, a data cleaning process was performed to remove incomplete surveys. No missing data or significant outliers were identified, so the full final sample was used for analysis. Regarding statistical assumptions, multivariate normality was not formally assessed, as the central limit theorem suggests that for samples larger than 30 participants, the sampling distribution of the estimators tends to be normal, allowing the application of models such as multiple linear regression without this assumption being strictly necessary [45]. For data processing and analysis, the collected data will be tabulated using SPSS statistical software, version 29. Descriptive analysis will then be conducted, utilizing measures of central tendency, such as mean and standard deviation. Inferential analysis will be conducted using Pearson’s correlation coefficient, Student’s t-test, one-way ANOVA, and the multiple linear regression model (using the stepwise input method). A significance level of 5% (0.05) will be applied to determine the statistical significance of the correlations and coefficients in the multiple linear regression model.

## Results

Table 2 provides descriptive and comparative analyses of BMI, general self-efficacy, and mental health based on the gender of the adolescents. Descriptive results indicate that, on average, BMI was slightly higher in girls (M = 22.88, SD = 4.15) compared to boys (M = 22.09, SD = 3.78). However, this difference was not statistically significant (*p* = 0.066) and the effect size was small (*d* = -0.20). With respect to general self-efficacy, boys (M = 34.76, SD = 6.72) and girls (M = 34.53, SD = 6.32) exhibited very similar scores, with no significant differences (*p* = 0.741) and a nearly negligible effect size (*d* = 0.04). Similarly, mental health scores were nearly identical between boys (M = 16.66, SD = 3.16) and girls (M = 16.90, SD = 3.41), with no significant difference (*p* = 0.515) and a very small effect size (*d* = -0.07).

**Table 2:** Descriptive and comparative analysis of BMI, self-efficacy and mental health according to gender of adolescents

	General (*n* = 343)		Male (*n* = 170)		Female (*n* = 173)		*t*	*p*	*d*
	M	SD	M	SD	M	SD			
**BMI**	22.49	3.99	22.09	3.78	22.88	4.15	-1.845	0.066	-0.20
**Self-efficacy**	34.65	6.51	34.76	6.72	34.53	6.32	0.331	0.741	0.04
**Mental health**	16.78	3.29	16.66	3.16	16.90	3.41	-0.651	0.515	-0.07

Note. M = Mean; SD = standard deviation; Student’s t-test statistic. *d* = d de Cohen (effect size)

Table 3 presents the descriptive and comparative analyses of BMI, self-efficacy, and mental health according to the age of the adolescents. The descriptive results indicate a slight increase in BMI with age, ranging from an average of 21.63 (SD = 4.22) at age 13 to 24.35 (SD = 2.53) at age 17. However, this variation was not statistically significant (F = 0.574, *p* = 0.682), and the effect size, as measured by Cohen’s f, was very small (*f* = 0.10). Regarding self-efficacy, a gradual increase in the mean was observed, from 33.76 (SD = 6.20) at age 13 to 38.00 (SD = 7.16) at age 17. However, this increase also did not reach statistical significance (F = 2.024, *p* = 0.091), and the effect size was small (*f* = 0.18). In terms of mental health, the means also showed an increase with age, from 16.24 (SD = 3.02) at age 13 to 17.62 (SD = 4.23) at age 17. However, these differences were not statistically significant (F = 2.266, *p* = 0.062), and the effect size was small (*f* = 0.20).

**Table 3:** Descriptive and comparative analyses of BMI, self-efficacy, and mental health were conducted based on the age of the adolescents

	Age					F	*p*	*f*
	13	14	15	16	17			
	M (SD)	M (SD)	M (SD)	M (SD)	M (SD)			
**BMI**	21.63 (4.22)	21.96 (3.70)	22.77 (4.04)	23.04 (4.17)	24.35 (2.53)	0.574	0.682	0.10
**Self-efficacy**	33.76 (6.20)	33.85 (5.68)	34.63 (7.14)	35.73 (6.59)	38.00 (7.16)	2.024	0.091	0.18
**Mental health**	16.24 (3.02)	16.81 (3.12)	16.88 (3.24)	16.82 (3.28)	17.62 (4.23)	2.266	0.062	0.20

Note. M = Mean; SD = standard deviation; One-way ANOVA statistical test; *f* = f de Cohen (effect size); 13 age (*n* = 50), 14 age (*n* = 101), 15 age (*n* = 102), 16 age (*n* = 77), 17 age (*n* = 13)

Table 4 presents the correlations between Body Mass Index (BMI), self-efficacy, and mental health among adolescents. The results reveal a significant negative correlation between BMI and mental health (*r* = -0.227, *p* < 0.001), suggesting that higher BMI is associated with lower levels of mental health in adolescents. Additionally, self-efficacy is positively correlated with mental health (*r* = 0.463, *p* < 0.001), indicating that higher self-efficacy is associated with better mental health.

**Table 4:** Correlation analysis of BMI, self-efficacy and adolescent mental health

	IMC	Self-efficacy	Mental health
**BMI**	1		
**Self-efficacy**	-0.055	1	
**Mental health**	-0.227^**^	0.463**	1

Note. **p < 0.01 (Moderately significant). The relationship analysis was done using Pearson’s correlation coefficient

The results of the multiple regression analysis (Table 5) indicate that Model 2, compared to Model 1, is statistically significant both overall (adjusted R² = 0.251, F = 58.295, *p* < 0.001) and in terms of individual coefficients (*p* < 0.001). The adjusted coefficient of determination (R² = 0.251) indicates that the predictor variables, BMI and self-efficacy, explain 25.1% of the variability in adolescent mental health. Furthermore, the ANOVA result (F = 58.295, *p* < 0.001) demonstrated a significant linear relationship between the predictor variables (BMI and self-efficacy) and the criterion variable (mental health). Similarly, the standardized coefficients (β) indicate that self-efficacy (β = 0.452) has a greater impact on the prediction of mental health than BMI (β = -0.202). In other words, self-efficacy is the most influential and relevant factor in predicting adolescent mental health compared to BMI.

**Table 5:** Multiple regression model

Model		Unstandardized coefficients		Standardized coefficients	*t*	*p*
		B	Standard Error	β		
1	**(Constant)**	10.805	2.280		4.739	< 0.001***
	**BMI**	0.226	0.024	0.448	9.454	< 0.001***
	**Self-efficacy**	-0.176	0.039	-0.214	-4.478	< 0.001***
	**Age**	0.100	0.145	0.033	0.685	0.494
	**Sex**	0.425	0.309	0.065	1.377	0.169
2	**(Constant)**	12.620	1.237		10.202	< 0.001***
	**BMI**	-0.167	0.039	-0.202	-4.310	< 0.001***
	**Self-efficacy**	0.228	0.024	0.452	9.648	< 0.001***

Note. Dependent variable: mental health; Model 1: *R*^2^*adjusted* = 0.260, ANOVA F (F = 29.757, *p* < 0.001); Model 2: *R*^2^*adjusted* = 0.251, ANOVA F (F = 58.295, *p* < 0.001). *** *p* < 0.001 (Highly significant)

## Discussion

Adolescent mental health has garnered increasing attention in recent decades due to its profound and lasting impact on the well-being and overall development of this population [46]. Among the factors that can influence mental health, BMI has received considerable attention due to its association with body perception, bullying, and other social factors that may trigger emotional problems [11, 17]. However, in parallel, self-efficacy-individuals’ belief in their ability to face challenges and achieve goals-is presented as a key protective psychological factor in promoting positive mental health [6, 23, 24, 26, 32, 33]. In this context, the present study aims to explore the predictive role of BMI and self-efficacy in the mental health of Peruvian adolescents. The primary findings reveal a statistically significant negative relationship between BMI and adolescent mental health. In contrast, self-efficacy was found to be positively correlated with mental health. Additionally, the multiple regression analysis revealed that both BMI and self-efficacy are significant predictors of mental health, with self-efficacy having a greater influence in the prediction. Together, these variables explain 25.1% (adjusted R²) of the variability in adolescent mental health.

High BMI is an increasing concern that affects not only the physical health but also the mental well-being of adolescents and the general population [8, 10, 11]. Adolescents who are overweight or obese often experience stigmatization, which increases their risk of developing mental health issues, such as anxiety, low self-esteem, and depression [11, 47]. The present study found a statistically significant relationship between BMI and adolescent mental health, supporting the hypothesis that physical fitness directly influences psychological well-being in this population [47]. Furthermore, the multiple regression analysis indicated that BMI is a significant predictor of mental health, suggesting that adolescents with higher BMI are more likely to experience emotional difficulties, such as anxiety and depression, compared to those with BMI values within the normal range. Furthermore, the multiple regression analysis indicated that BMI is a significant predictor of mental health, suggesting that adolescents with higher BMI are more likely to experience emotional difficulties, such as anxiety and depression, compared to those with BMI values within the normal range [11].

Our findings align with previous research that has demonstrated a negative relationship between elevated BMI and mental health in adolescents [11, 19, 48, 49]. For example, Rostampour et al., [48] showed a significant correlation between BMI and mental health problems (depression, anxiety) in Iranian adolescents. Similarly, Nauli et al. [19] confirmed the relationship between BMI and mental health among Islamic adolescents. Likewise, Ocampo et al. [49] demonstrated that higher BMI in adolescent females is associated with an increased risk of depression. Additionally, complementary studies, such as that of Lindberg et al. [50], suggest that obesity is a significant risk factor for the development of anxiety and depression in children and adolescents. On the other hand, it is important to note that the relationship between obesity and mental health may be influenced by various factors, including shared environmental, physiological, and genetic components, as well as adolescents’ perception of their weight status [51]. For example, in a recent study, girls who perceived themselves as overweight—regardless of their actual body weight—or who overestimated their weight were more likely to experience depressed mood and stress. In contrast, boys who perceived themselves as underweight were more likely to experience suicidal ideation compared to those with average weight perception or an accurate understanding of their weight status [11].

Additionally, obesity is linked to metabolic and hormonal alterations that may affect mood regulation. Elevated levels of chronic inflammation and insulin resistance, both common in individuals with obesity, have been implicated in the pathophysiology of depression and anxiety [52]. Furthermore, imbalances in neurotransmitters such as serotonin and dopamine, which are involved in both appetite and mood regulation, may exacerbate the comorbidity between obesity and mental disorders [53]. Several additional factors may contribute to the relationship between obesity and mental health. Key among these are a sedentary lifestyle, an imbalanced diet, and sleep-related problems, all of which have been associated with an increased risk of both depression and obesity [52, 54] [46, 48]. In addition, sleep problems, such as insomnia or sleep apnea, which are common in overweight people, can further aggravate the cycle of obesity and deteriorating mental health [55]. Sleep deprivation disrupts appetite control mechanisms, increases stress levels, and has been associated with higher incidences of anxiety and depression [56]. Therefore, early interventions should not only address obesity from a physical perspective, but also focus on promoting emotional well-being and developing coping skills to manage social pressure.

The existing literature consistently underscores the importance of self-efficacy as a crucial protective factor for adolescent mental health [6, 23, 24, 26, 32, 33]. The positive impact of self-efficacy on stress management, emotion regulation, and coping with external challenges suggests that interventions aimed at enhancing self-efficacy may be effective in promoting psychological well-being in this vulnerable population [23, 24]. In our study, self-efficacy was found to be positively and significantly correlated with adolescent mental health. This suggests that adolescents who have confidence in their ability to manage challenging situations and achieve their goals tend to experience higher levels of psychological well-being. Additionally, multiple regression analysis indicated that self-efficacy is a significant predictor of mental health, suggesting that developing skills and self-confidence may be essential in protecting adolescents from mental disorders such as anxiety and depression. Previous studies further support the relationship between self-efficacy and mental health [6, 23, 24, 26, 32, 33]. For example, Zhang et al., [23] showed a significant relationship between self-efficacy and mental health among adolescents in China. Similarly, Antony et al., [6] confirmed the correlation between self-efficacy and mental health in adolescents in India. Moreover, complementary studies suggest that adolescents with higher self-efficacy demonstrate better emotional regulation and greater resilience to stress [57]. In fact, Tahmassian et al. [57] noted that self-efficacy not only enhances the emotional well-being of adolescents but also reduces the likelihood of developing symptoms of anxiety and depression. This connection has also been observed in other contexts, with research suggesting that self-efficacy serves as a buffer against the negative effects of social and academic stress, enabling adolescents to better cope with external pressures and maintain more stable mental health [58]. These findings underscore the importance of fostering self-efficacy in educational and mental health programs, as its promotion could directly impact the emotional well-being of young people.

Notably, in the present study, self-efficacy demonstrated a greater predictive value for mental health compared to BMI, suggesting that psychological factors may have a more direct influence on the emotional well-being of adolescents than physical factors. This finding aligns with previous research that identifies self-efficacy as a key determinant of mental health, due to its role in emotional regulation, stress management, and individuals’ capacity to cope with challenging situations [23, 24]. While BMI, as a physical indicator, is linked to mental health through indirect mechanisms such as social stigmatization or low self-esteem, self-efficacy seems to exert a more robust protective effect by instilling confidence in adolescents to overcome everyday challenges [22]. Therefore, it is essential to approach adolescent mental health from a multifactorial perspective that considers not only physical health but also psychological beliefs and skills. In other words, while elevated BMI may be associated with higher levels of depression or anxiety [11, 19], an adolescent’s belief in their ability to manage these challenges can help mitigate the negative effects of being overweight on their emotional well-being [6, 23, 24, 26, 32, 33].

Finally, in the present study, it was observed that BMI and self-efficacy together account for 25.1% of the variability in adolescent mental health. While both variables are important, it is likely that other factors not considered in this model also contribute to the mental health of this population. It is important to acknowledge that mental health is a multifaceted construct influenced by a wide range of factors, including biological, social, familial, and contextual aspects, which were not addressed in the present study [59]. Although the proportion explained by these two variables is significant, it suggests that BMI and self-efficacy are not the only relevant predictors. Other studies have found that factors such as social support, family relationships, academic stress, and sleep quality also have a substantial impact on the mental health of adolescents and the general population [60, 61]. The fact that BMI and self-efficacy account for a quarter of the variability suggests that promoting healthy behaviors related to self-efficacy, alongside attention to physical health, may be an effective strategy for improving mental health during this stage of life [59]. However, to maximize the effectiveness of interventions, it is essential to incorporate other critical dimensions, such as the development of socioemotional skills and the creation of supportive environments within both family and school settings, as these factors have been shown to strongly influence the psychological well-being of adolescents [62].

### Public health implications

The results of this study have important public health implications, particularly for the design of interventions aimed at improving adolescent mental health. Firstly, the findings suggest that programs focused on enhancing adolescents’ confidence in their ability to cope with challenges could positively impact their emotional well-being. Strategies focused on developing coping, stress management, and problem-solving skills should be prioritized in both school and community settings. Promoting self-efficacy through educational and psychological activities could not only reduce the risk of disorders such as anxiety and depression but also empower adolescents to make healthier choices in other areas of their lives. Secondly, adolescent obesity should be addressed from a holistic perspective that extends beyond physical interventions. Public health campaigns should incorporate components that not only promote healthy eating habits and physical activity but also address the psychological impacts of obesity, such as social stigma and self-esteem issues. The creation of supportive, stigma-free environments in schools and communities could facilitate better integration of overweight adolescents, leading to improvements in their mental health. Thirdly, the headspace model in Australia represents a valuable reference for the Peruvian context. This approach offers comprehensive care for adolescents, combining mental, physical, sexual, and vocational services in a single space, with high accessibility and positive results. Based on early intervention, youth participation, and multidisciplinary teams, this model could be adapted to the Peruvian context, specifically considering the variables studied in this research, such as body mass index (BMI) and self-efficacy, with the aim of improving the mental health of adolescents [63].

Finally, the fact that both variables together account for 25.1% of the variability in adolescent mental health suggests that public health intervention programs should adopt a comprehensive approach. This entails not only addressing physical aspects, such as weight, but also promoting psychological well-being by fostering socioemotional skills, creating supportive social and family environments, and reducing risk factors associated with stress and isolation. Public health policies targeting adolescents should be multifaceted, addressing both personal factors, such as self-efficacy, and social and physical factors that impact their well-being.

### Limitations and future prospects

Despite the relevant findings of this study, it is important to point out some limitations and propose recommendations for future research. Regarding the methodological design, the cross-sectional approach limits the possibility of establishing causal relationships between the variables analyzed. Therefore, it is not possible to determine with certainty whether self-efficacy and BMI directly influence the mental health of adolescents, or whether there are other factors not considered that also have a significant effect. In this regard, it is recommended that future studies adopt a longitudinal design, which would allow for a more accurate analysis of the temporal and causal relationships between these variables.

With regard to the sample, as it was selected from a single educational institution located in Metropolitan Lima, a limitation in terms of geographical and sociocultural diversity is acknowledged. This characteristic could have introduced bias into the findings, given that the results may be conditioned by factors specific to the educational, economic, or family environment of the participants and may not be representative of the Peruvian adolescent population in general. Therefore, it is recommended that future research include larger and more representative samples from different regions of the country in order to improve the generalizability of the results and explore possible contextual, cultural, regional, or socioeconomic differences. Furthermore, considering that multiple statistical analyses were performed in this study, it is suggested that these findings be replicated in other samples to confirm the robustness and consistency of the results obtained.

Specifically, it is also suggested that the possible relationship between BMI and self-efficacy be examined, given that these variables may not be completely independent of each other. In this regard, it would be relevant to assess whether self-efficacy acts as a moderating variable in the relationship between BMI and mental health. Furthermore, sensitivity analyses are recommended to assess the stability and consistency of the results, especially when applying the models to different subgroups or control conditions. Furthermore, future research should analyze nutritional status as such, using age- and sex-adjusted BMI (Z scores) according to WHO growth curves, rather than crude BMI, in order to compare results and explore whether this methodological distinction produces differences in the associations observed with mental health. Furthermore, although this study has suggested that higher BMI and lower self-efficacy may predict poorer mental health, it is also important to consider the reverse possibility: that poorer mental health may negatively influence BMI and self-efficacy scores.

Finally, although the study examined both BMI and self-efficacy, other factors related to physical well-being, such as physical activity and diet quality, could offer a more comprehensive understanding of the determinants of mental health in adolescents. Including these variables in future research would aid in developing more thorough and targeted interventions to enhance the well-being of young people. Looking ahead, future research should explore the specific mechanisms through which self-efficacy influences mental health, as well as the mediating or moderating roles of additional variables, such as social environment and emotional support. Additionally, implementing intervention studies that assess the impact of programs designed to enhance self-efficacy and improve adolescent mental health could have significant implications for public health policies.

## Conclusion

The findings of this study revealed that both self-efficacy and BMI are significant predictors of mental health in adolescents, with self-efficacy demonstrating a greater influence compared to BMI. These results not only deepen our understanding of the determinants of mental health in adolescents but also highlight the importance of fostering self-efficacy in public health interventions aimed at improving mental health within this population. These results not only deepen our understanding of the determinants of mental health in adolescents but also highlight the importance of fostering self-efficacy in public health interventions aimed at improving mental health within this population.

## Data Availability

No datasets were generated or analysed during the current study.

## Abbreviations

ANOVA: Analysis of Variance
BMI: Body Mass Index
CI: Confidence Interval
*d*: Cohen’s d
*f*: Cohen’s f
GSE: General Self-Efficacy Scale
R-MHI-5: Mental Health Inventory-5
SD: Standard Deviation
SPSS: Statistical Package for the Social Sciences
WHO: World Health Organization
